# Brassinolide Improves the Tolerance of *Hydrilla verticillata* to Low-Temperature Stress

**DOI:** 10.3390/biology15100783

**Published:** 2026-05-14

**Authors:** Yuhan Zhu, Jingwen Wang, Meiqin Wu, Peimin He, Liu Shao, Jinlin Liu

**Affiliations:** 1 College of Oceanography and Ecological Science, Shanghai Ocean University, Shanghai 201306, China; 17851618619@163.com (Y.Z.); 13382079120@163.com (J.W.); mqwu@shou.edu.cn (M.W.); pmhe@shou.edu.cn (P.H.); 2 Water Environment and Ecology Engineering Research Center of the Shanghai Institution of Higher Education, Shanghai Ocean University, Shanghai 201306, China; 3 Key Laboratory of Marine Ecological Monitoring and Restoration Technologies, East China Sea Ecological Center, Ministry of Natural Resources, Shanghai 201206, China; 4 Project Management Office of China National Scientific Seafloor Observatory, Tongji University, Shanghai 200092, China

**Keywords:** *Hydrilla verticillata*, brassinolide, low-temperature stress, physiological mechanisms

## Abstract

Low temperature severely constrains the growth and ecological application of submerged macrophytes in aquatic ecosystem restoration. Although brassinolide (BR) has been shown to alleviate abiotic stress in terrestrial plants, its role in submerged macrophytes remains poorly understood. This study investigated the effects of different BR concentrations (0, 0.05, 0.1, and 0.5 mg L^−1^) on growth, photosynthesis, antioxidant defense, and osmotic adjustment in *Hydrilla verticillata* under low-temperature stress (2 °C) over 15 days. Exogenous BR significantly alleviated low-temperature-induced damage in *H. verticillata* in a concentration-dependent manner. Among all treatments, 0.05 mg L^−1^ BR showed the strongest overall effect, increasing fresh weight, dry weight, and plant height by 16.22%, 22.67%, and 9.52%, respectively, compared with the control. It also promoted photosynthetic performance, with *Fv*/*Fm* and Y(II) increasing by 251.93% and 262.83%, respectively, on day 10, and enhanced stress resistance, as reflected by a 32.44% increase in SOD activity and a 112.59% increase in soluble sugar content on day 15. In contrast, higher BR concentrations (0.1 and 0.5 mg L^−1^) were less effective overall. Membership function analysis ranked the treatments as 0.05 mg L^−1^ (0.95) > 0.1 mg L^−1^ (0.47) > 0.5 mg L^−1^ (0.19) > 0 mg L^−1^ (0.09). These results indicate that BR enhances low-temperature tolerance in *H. verticillata* through coordinated regulation of photosynthetic performance, antioxidant defense, and osmotic adjustment, providing a physiological basis for its application in submerged macrophyte restoration under low-temperature conditions.

## Simple Summary

Low temperatures significantly hinder the growth of submerged macrophytes, which are essential for restoring degraded aquatic ecosystems. Although brassinolide (BR), a natural plant hormone, has been shown to enhance stress tolerance in terrestrial plants, its role in submerged macrophytes remains unclear. This study examined the effects of exogenous BR on the low-temperature tolerance of *Hydrilla verticillata*, a common submerged macrophyte, under 15 days of low-temperature stress at 2 °C. Plants were treated with four different BR concentrations to identify the most effective concentration for promoting overwinter survival. The results showed that BR significantly enhanced the low-temperature tolerance of *H. verticillata*, with 0.05 mg L^−1^ producing the best overall performance. Compared with untreated plants, this treatment better maintained growth status and photosynthetic performance. It also increased antioxidant enzyme activities and the accumulation of osmotic adjustment substances, which help protect cells from low-temperature-induced damage. Although higher BR concentrations also showed some protective effects, their overall performance was inferior to that of the lower concentration. These findings indicate that BR has strong potential for the winter conservation of submerged macrophytes and for aquatic ecological restoration.

## 1. Introduction

Temperature is a key environmental factor that regulates plant growth, development, and ecological adaptation [1]. Low-temperature stress can induce a series of physiological and biochemical disturbances in plants [2]. It disrupts chloroplast ultrastructure and inhibits the activity of key enzymes involved in chlorophyll biosynthesis, thereby impairing chlorophyll accumulation, chloroplast development, and photosynthetic efficiency, ultimately suppressing plant growth [3,4]. In addition, low temperature can promote the excessive accumulation of reactive oxygen species (ROS), resulting in membrane lipid peroxidation, cellular damage, and the accumulation of malondialdehyde (MDA), a typical indicator of oxidative injury [5]. To alleviate low-temperature-induced damage, plants activate multiple defense mechanisms to maintain cellular integrity and physiological activity [6]. The antioxidant defense system is one of the major protective pathways involved in plant responses to low-temperature stress. Excess ROS can be scavenged through the coordinated action of antioxidant enzymes, among which superoxide dismutase (SOD), peroxidase (POD), and catalase (CAT) play important roles in free radical scavenging, oxidative damage mitigation, and the enhancement of low-temperature tolerance [7,8]. Meanwhile, osmotic adjustment substances help maintain cellular osmotic balance and water status, thereby reducing dehydration and membrane injury under low-temperature conditions. Soluble protein (SP), soluble sugar (SS), and proline (Pro) are common osmolytes that contribute to plant resistance to low-temperature stress [5,9].

In the ecological restoration of lakes and other degraded aquatic ecosystems, the re-establishment of submerged macrophytes is widely regarded as an effective strategy for restoring ecosystem structure and function [10,11]. *Hydrilla verticillata*, a typical submerged macrophyte broadly distributed in freshwater ecosystems, plays important ecological roles in water purification, nutrient removal, sediment stabilization, and the provision of habitats for aquatic organisms [12,13]. Owing to these functions, *H. verticillata* has been widely used in lake management, aquatic ecological restoration, and submerged vegetation recovery. Although *H. verticillata* is generally considered to have relatively strong environmental adaptability, its growth and ecological functions are highly temperature-dependent. Low temperatures can significantly affect the growth, overwintering performance, and distribution of submerged macrophytes [14]. The optimal growth temperature for *H. verticillata* is generally 28–32 °C, whereas its growth is severely inhibited at temperatures of 16 °C or lower [15]. Such growth inhibition is not only reflected in reduced biomass accumulation and photosynthetic performance, but may also weaken its ecological functions, particularly its capacity for water purification and nutrient removal. Previous studies have shown that temperature markedly affects the nutrient removal efficiency of *H. verticillata*, with nutrient removal under low-temperature conditions being only approximately half of that observed under more suitable temperature conditions [16]. In many regions, winter and early-spring low temperatures may limit the active growth of *H. verticillata* and consequently reduce its ecological function and purification capacity in aquatic restoration systems. Accordingly, investigating the physiological responses of *H. verticillata* to low-temperature stress and identifying effective measures to improve its low-temperature tolerance are necessary.

Brassinolide (BR), an important steroidal plant hormone, plays a central role in the regulation of plant growth, development, and stress resistance [17]. Previous studies have shown that BR can alleviate the adverse effects of low temperature, salinity, and drought by enhancing antioxidant capacity, promoting chlorophyll accumulation, and maintaining membrane stability in terrestrial plants, such as grapes (*Vitis* spp.), rice (*Oryza sativa*), and barley (*Hordeum vulgare*) [18,19,20]. In addition to terrestrial plants, BR has shown promising physiological effects in aquatic photoautotrophic organisms. BR application has been reported to promote biomass accumulation, organic compound production, cell proliferation, and metabolic enzyme activities in *Dunaliella parva*, *Acutodesmus obliquus*, and *Asterionella formosa* [21,22,23]. More directly, studies on submerged macrophytes have shown that BR exposure through immersion treatment at an appropriate concentration and duration can promote growth, enhance reproductive capacity, and increase chlorophyll accumulation in *Vallisneria natans* [24]. These findings suggest that BR can regulate growth and physiological performance in aquatic plant systems. However, studies on BR-mediated abiotic stress tolerance in submerged macrophytes remain limited. Given their distinctive aquatic habitat, submerged macrophytes may exhibit species-specific responses to BR in terms of effective concentration range, duration of action, uptake pattern, and physiological regulation. Therefore, further investigation is needed to clarify the regulatory effects and underlying mechanisms of BR in submerged macrophytes under abiotic stress conditions.

Based on this background, this study investigated the effects of different BR concentrations on growth, photosynthetic performance, antioxidant enzyme activities, and osmotic adjustment in *H. verticillata* under low-temperature stress. The objectives were to determine whether exogenous BR could alleviate low-temperature-induced physiological damage, identify an effective BR concentration, and clarify the physiological basis of BR-mediated low-temperature tolerance in submerged macrophytes. These findings may provide a theoretical foundation and technical support for the construction of submerged macrophyte communities and the ecological restoration of aquatic ecosystems under low-temperature conditions.

## 2. Materials and Methods

### 2.1. Experimental Materials and Pretreatment

*H. verticillata* was used as the experimental material in this study. Healthy plants with uniform morphology were collected from the Fengjing Base of Shanghai Taihe Water Environment Technology Co., Ltd. (Jinshan District, Shanghai, China). Before the experiment, plant surfaces were gently rinsed with ultrapure water to remove attached debris and epiphytes.

To enhance acclimation to low-temperature conditions, *H. verticillata* was placed in plastic containers filled with filtered water for 7 days prior to the experiment. During this acclimation period, the temperature in the growth chamber was gradually decreased from 25 °C to 2 °C at a rate of 3–4 °C per day. Subsequently, BR treatment experiments were conducted under low-temperature stress at 2 °C. Illumination was provided by LED lamps (Yee, Qingdao, China) at a light intensity of 100 μmol m^−2^ s^−1^ under a 12 h light/12 h dark photoperiod.

BR was purchased from Shanghai Yuanye Bio-Technology Co., Ltd (Shanghai, China). Briefly, 10 mg of BR was first dissolved in a small volume of dimethyl sulfoxide (DMSO), and the final volume was adjusted with ultrapure water to obtain a 40 mg L^−1^ stock solution. Working solutions of 0.05, 0.1, and 0.5 mg L^−1^ were subsequently prepared by serial dilution of the stock solution with ultrapure water. All solutions were stored at 4 °C in the dark until use. Twelve hours before plant introduction, BR solutions at the designated concentrations were added to the corresponding beakers, which were then placed in a low-temperature incubator for pre-equilibration.

Experimental water was collected from a river on the Shanghai Ocean University campus and pre-filtered through 0.45 µm glass-fiber filters (Xinya, Shanghai, China). The filtered water was used for plant cultivation and subsequent low-temperature treatments.

### 2.2. Experimental Design

A hydroponic system was used in this study. After low-temperature acclimation, apical segments excised from the main stems of *H. verticillata* were used as experimental materials and trimmed to a uniform size, with a fresh weight of 1.50 ± 0.02 g and a length of 10.0 ± 0.1 cm. The trimmed plants were then transferred into 2 L beakers containing filtered water, with 30 plants in each beaker. Plants were fully immersed in the corresponding BR working solutions to ensure direct and uniform exposure to the treatment.

Based on the results of a preliminary dose–response experiment, four BR treatments were established under 2 °C low-temperature stress: 0 mg L^−1^ (control), 0.05 mg L^−1^, 0.1 mg L^−1^, and 0.5 mg L^−1^. During the treatment period, the BR-containing water was maintained at 2 °C in the growth chamber. Each treatment consisted of three replicate beakers, resulting in a total of 12 experimental units. During the treatment period, illumination was provided by LED lamps at 100 μmol m^−2^ s^−1^ under a 12 h light/12 h dark photoperiod.

The experiment lasted for 15 days. Fresh weight and dry weight were measured on days 0 and 15. Plant height was determined on days 0, 5, 10, and 15. Chlorophyll content, chlorophyll fluorescence parameters, MDA content, antioxidant enzyme activities, and osmotic adjustment substance contents were measured on days 0, 2, 5, 10, and 15. For all physiological and biochemical assays, fresh apical tissues were collected from the main stem segments to ensure uniform tissue age and size among treatments.

### 2.3. Determination of Fresh Weight, Dry Weight, and Plant Height

Surface moisture was gently removed from the plants with absorbent paper, and fresh weight (FW) was measured using an electronic balance. Plant samples were then dried in an oven at 80 °C for 48 h to constant weight, and the dry weight (DW) was recorded [25]. The biomass growth rate (BGR, %) was calculated as

$$\mathrm{BGR}\;(\%)=\frac{W_{2}-W_{1}}{W_{1}}\times 100$$

where *W*_1_ and *W*_2_ are the initial and final weights (g) of the macrophytes, respectively.

Plant height was measured with a ruler and expressed as the vertical distance from the basal end of the stem segment to the apical tip. The plant height growth rate (HGR, %) was calculated as

$$\mathrm{HGR}\;(\%)=\frac{H_{2}-H_{1}}{H_{1}}\times 100$$

where *H*_1_ is the initial plant height (10 cm), and *H*_2_ is the plant height measured on the sampling day.

### 2.4. Determination of Chlorophyll Content and Chlorophyll Fluorescence Parameters

Chlorophyll content was determined using 95% ethanol extraction according to the method described by Ogo et al. [26]. For chlorophyll analysis, 0.10 g of fresh apical tissue from the main stem was collected to ensure uniform tissue age and size. Samples were rinsed three times with ultrapure water, and surface moisture was gently removed with absorbent paper before the samples were transferred into a 10 mL amber centrifuge tube. Under dark conditions, 10 mL of 95% ethanol was added, and the tissue was mechanically homogenized. The homogenate was then extracted at 4 °C for 24 h. After extraction, the solution was centrifuged at 4000 rpm for 10 min, and the supernatant was collected. The absorbance (A) of the supernatant was measured at 649 and 665 nm using a UV-1800 UV-Vis spectrophotometer (Shimadzu Corporation, Kyoto, Japan), and chlorophyll a (Chl *a*), chlorophyll b (Chl *b*), and chlorophyll *a* + *b* (Chl *a* + *b*) contents were calculated using the following equations:

$$\mathrm{Chl}a\;(\mathrm{mg}g^{-1}\mathrm{FW})=(13.95\times A_{665}-6.88\times A_{649})\times V_{t}/\mathrm{FW}\times 1000$$

$$\mathrm{Chl}b\;(\mathrm{mg}g^{-1}\mathrm{FW})=(24.96\times A_{649}-7.32\times A_{665})\times V_{t}/\mathrm{FW}\times 1000$$

$$\mathrm{Chl}a+b\;(\mathrm{mg}g^{-1}\mathrm{FW})=(6.63\times A_{665}+18.08\times A_{649})\times V_{t}/\mathrm{FW}\times 1000$$

where *V*_t_ is the total extraction volume (mL), and FW is the fresh weight of the sample (g).

Chlorophyll fluorescence parameters were measured using a DUAL-PAM-100 dual-channel modulated chlorophyll fluorometer (Heinz Walz GmbH, Effeltrich, Germany). The maximum quantum yield of PSII (*Fv*/*Fm*) and the effective quantum yield of PSII [Y(II)] were recorded.

### 2.5. Determination of MDA Content and Antioxidant Enzyme Activities

MDA content, SOD activity, and POD activity were determined using the corresponding assay kits (Nanjing Jiancheng Bioengineering Institute, Nanjing, China) according to the manufacturer’s instructions. Approximately 0.10 g of fresh apical tissue from the main stem was homogenized with the appropriate extraction solution according to each assay protocol using a cryogenic grinder. The homogenates were centrifuged at 4 °C for 10 min at the speeds specified in the respective kit protocols, and the supernatants were collected for subsequent analysis. MDA content was determined using the thiobarbituric acid (TBA) method [27]. MDA reacted with TBA to form a red-colored product, and the absorbance was measured at 530 nm. SOD activity was determined using the hydroxylamine method [28]. Superoxide anion radicals (O_2_^−^) generated by the xanthine and xanthine oxidase system oxidized hydroxylamine to nitrite, which then reacted with the chromogenic reagent to form a purple-red complex. Absorbance was measured at 520 nm, and SOD activity was calculated accordingly. POD activity was determined using the guaiacol method [29]. POD activity was calculated from the change in absorbance at 420 nm based on the POD-catalyzed reaction of H_2_O_2_.

### 2.6. Determination of Soluble Protein and Soluble Sugar Contents

SP and SS contents were determined using the corresponding assay kits (Nanjing Jiancheng Bioengineering Institute, Nanjing, China) according to the manufacturer’s instructions. For SP determination, 0.10 g of fresh apical tissue from the main stem was homogenized in 0.1 mol L^−1^ phosphate buffer (pH 7.0–7.4) at a tissue-to-buffer ratio of 1:9 (g:mL) using a cryogenic grinder. The homogenate was centrifuged at 2500 rpm for 10 min at 4 °C, and the supernatant was collected for analysis. SP content was determined by the Coomassie Brilliant Blue method [30]. In this assay, the anionic dye binds to protein NH_3_^+^ groups to form a blue complex, and the absorbance was measured at 595 nm. For SS determination, 0.10 g of fresh apical tissue from the main stem was homogenized with distilled water at a sample-to-water ratio of 1:10 (g:mL). The homogenate was transferred to capped centrifuge tubes, heated in a boiling water bath for 10 min, and then cooled to room temperature. After centrifugation at 4000 rpm for 10 min at room temperature, the supernatant was collected and diluted 10-fold with distilled water. SS content was determined by the anthrone method [31]. Under concentrated sulfuric acid conditions, soluble sugars react with anthrone to form blue-green furfural derivatives, and the absorbance was measured at 620 nm.

### 2.7. Comprehensive Evaluation Based on Membership Function Values

A comprehensive evaluation method based on membership function values (MFV) was used to assess the alleviating effects of different BR concentrations [32]. For indicators positively associated with low-temperature tolerance, MFV was calculated as

$$\mu (X_{i})=\frac{X_{i}-X_{\min}}{X_{\max}-X_{\min}}$$

For indicators negatively associated with low-temperature tolerance, MFV was calculated as

$$\mu (X_{i})=\frac{X_{\max}-X_{i}}{X_{\max}-X_{\min}}$$

where *X_i_* is the observed value of a given indicator, *X*_min_ and *X*_max_ are the minimum and maximum values of that indicator among all treatments, respectively.

Furthermore, the overall membership function value (overall MFV) for each BR concentration was obtained by calculating the arithmetic mean of the MFVs of all evaluated indicators, as follows:

$$\mathrm{overall}\;\mathrm{MFV}=\frac{1}{n}\sum_{i=1}^{n}{\mathrm{MFV}}_{i}$$

where *n* is the total number of evaluated indicators, and MFV*_i_* is the membership function value of the *i*th indicator.

### 2.8. Statistical Analysis

All data are expressed as the mean ± standard deviation (SD) of three independent biological replicates. Statistical analyses were performed using IBM SPSS Statistics 27.0 (IBM Corp., Armonk, NY, USA). After testing for normality and homogeneity of variance, differences among BR treatments at each sampling time were evaluated using one-way analysis of variance (ANOVA), followed by Tukey’s multiple comparison test. Differences were considered statistically significant at *p* < 0.05. All figures were prepared using OriginPro 2024 (OriginLab Corp., Northampton, MA, USA).

## 3. Results

### 3.1. Effect of Different BR Concentrations on Biomass and Plant Height

**Figure 1:**
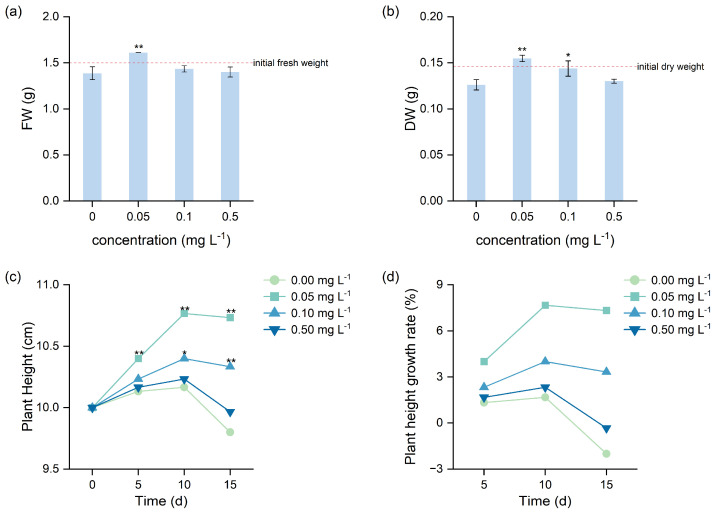
Effects of BR on fresh weight (**a**), dry weight (**b**), plant height (**c**) and plant height growth rate (**d**) of *H. verticillata* under 2 °C low-temperature stress. * indicates a significant difference compared with the control at *p* < 0.05, and ** indicates a highly significant difference compared with the control at *p* < 0.01.

Under low-temperature stress, different BR concentrations exerted distinct effects on biomass accumulation and plant height in *H. verticillata*, with the 0.05 mg L^−1^ treatment showing the most pronounced promotive effect (Figure 1). After 15 days of low-temperature treatment, fresh weight was significantly increased only in the 0.05 mg L^−1^ BR treatment (*p* < 0.01), representing a 16.22% increase compared with the control and a 7.41% increase relative to the initial value (Figure 1a; Table 1). In contrast, 0.1 and 0.5 mg L^−1^ treatments resulted in only slight increases of 3.36% and 1.04%, respectively, relative to the control, although these differences were not statistically significant. The enhancement in dry weight was more pronounced. The 0.05 mg L^−1^ BR treatment significantly increased dry weight (*p* < 0.01), with increases of 22.67% relative to the control and 5.93% relative to the initial value. The 0.1 mg L^−1^ treatment also caused a significant increase (*p* < 0.05), with a 13.98% gain compared with the control. The 0.5 mg L^−1^ treatment did not differ significantly from the control (Figure 1b).

Plant height showed an initial increase followed by a decline under low-temperature stress, while exogenous BR partially promoted plant elongation and delayed the subsequent decline (Figure 1c). In the control, plant height decreased to 9.80 ± 0.10 cm by day 15, representing a 2% reduction compared with the initial value. The 0.05 mg L^−1^ BR treatment significantly increased plant height at all sampling time points, with increases of 4.00%, 7.67%, and 7.33% relative to the initial value on days 5, 10, and 15, respectively (Figure 1d), and increases of 2.63%, 5.90%, and 9.52% compared with the control (*p* < 0.01). The 0.1 mg L^−1^ treatment had no significant effect during the early stage but enhanced growth during the middle and late stages, showing increases of 4.00% and 3.33% relative to the initial value on days 10 and 15, respectively, and increases of 2.30% and 5.44% compared with the control. The 0.5 mg L^−1^ treatment showed only a weak promotive effect, which was not statistically significant.

### 3.2. Effect of Different BR Concentrations on Chlorophyll Content and Chlorophyll Fluorescence Parameters

**Figure 2:**
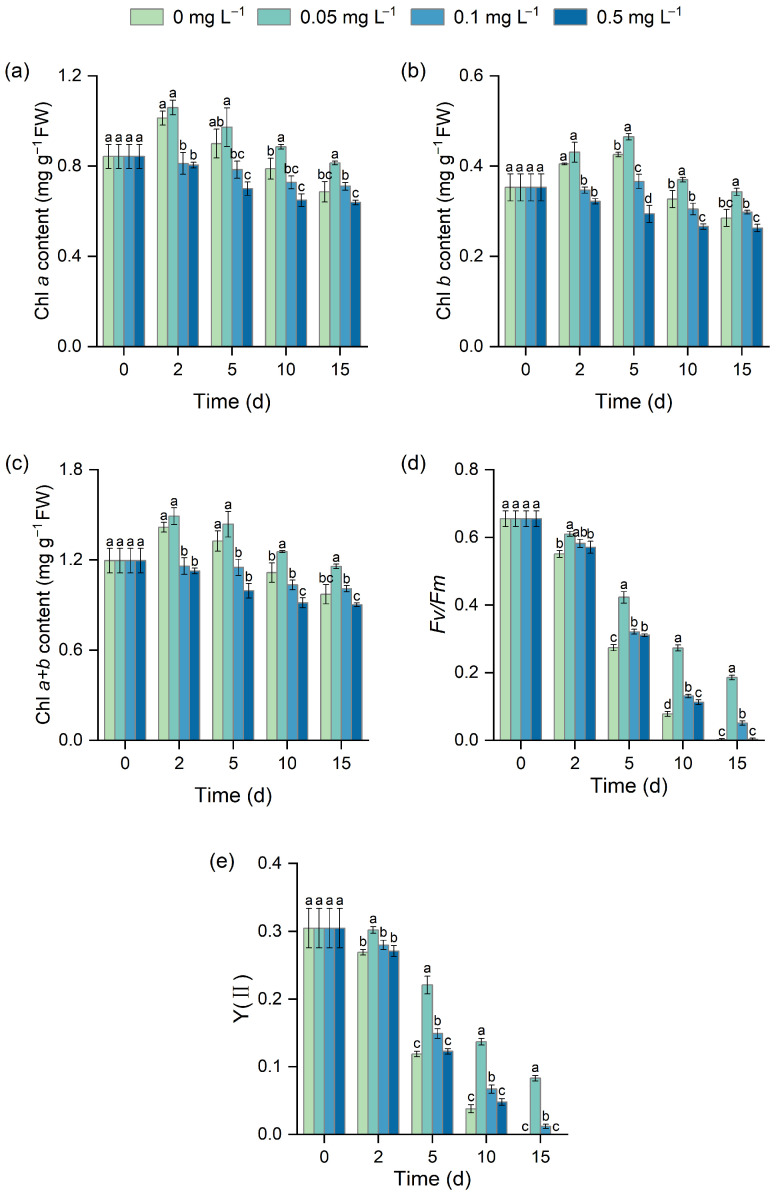
Effects of BR on Chl *a* (**a**), Chl *b* (**b**), Chl *a* + *b* (**c**), *Fv*/*Fm* (**d**), and Y(II) (**e**) in *H. verticillata* under 2 °C low-temperature stress. Different letters indicate significant differences among concentration treatments on the same sampling day (*p* < 0.05).

Chlorophyll content was significantly affected by BR under low-temperature stress (Figure 2a–c). On day 2, none of the BR treatments significantly increased Chl *a*, Chl *b*, or Chl *a* + *b*, and the medium and high concentrations showed slight inhibitory trendencies. During the middle and late stages of stress, the 0.05 mg L^−1^ BR treatment exhibited a relatively stable promotive effect. Chl *a* increased significantly on day 10, showing a 12.30% gain compared with the control (Figure 2a), while Chl b increased significantly on day 5 by 9.15% relative to the control (Figure 2b). By day 15, the 0.05 mg L^−1^ treatment further promoted chlorophyll accumulation, with Chl *a*, Chl *b*, and Chl *a* + *b* increasing by 18.66%, 20.37%, and 19.16% (*p* < 0.05), respectively. In contrast, treatments with 0.1 mg L^−1^ and 0.5 mg L^−1^ did not show significant enhancement.

*Fv*/*Fm* and Y(II) decreased gradually under low-temperature stress, while BR partially alleviated this decline (Figure 2d,e). On day 2, only 0.05 mg L^−1^ BR significantly increased *Fv*/*Fm* by 10.58% relative to the control (*p* < 0.05). During the middle stage of stress, the three BR concentrations significantly enhanced *Fv*/*Fm*. Among them, 0.05 mg L^−1^ BR exhibited the greatest protective effect on day 10, with an increase of 251.93% compared with the control. By day 15, *Fv*/*Fm* in the control had decreased to zero, whereas the 0.05 mg L^−1^ BR treatment still maintained a value of 0.19 ± 0.01 (Figure 2d). Y(II) responded similarly, with 0.05 mg L^−1^ showing the strongest alleviating effect. On days 2, 5, and 10, Y(II) increased by 12.14%, 84.92%, and 262.83%, respectively, compared with the control (*p* < 0.05). The 0.1 mg L^−1^ BR treatment also had a positive effect on Y(II), increasing the values by 24.58% and 78.76% compared with the control on days 5 and 10, respectively. In contrast, the 0.5 mg L^−1^ BR treatment had no significant effect (Figure 2e).

### 3.3. Effect of Different BR Concentrations on MDA Content and Antioxidant Enzyme Activities

**Figure 3:**
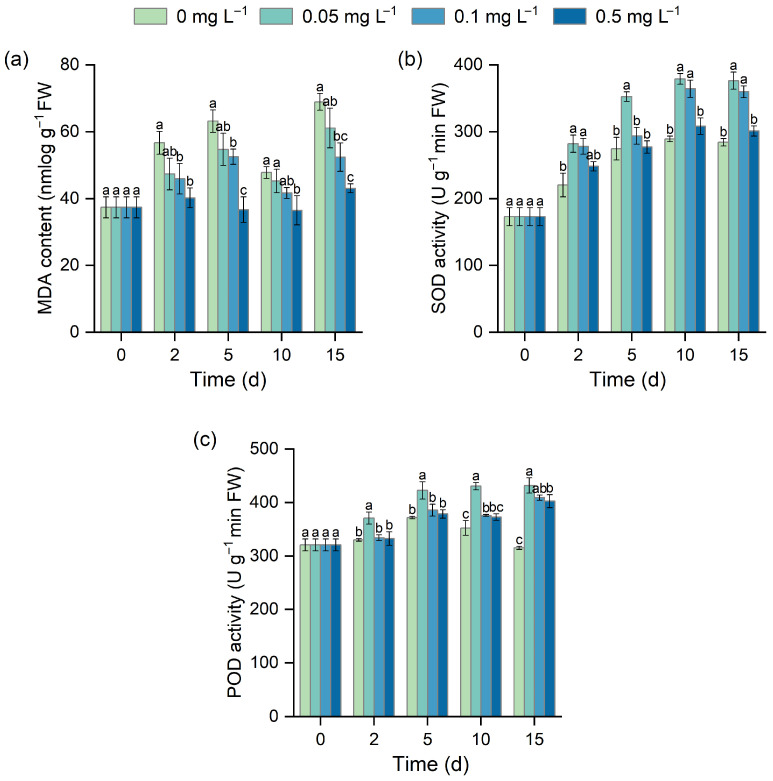
Effects of BR on MDA content (**a**), SOD activity (**b**), and POD activity (**c**) in *H. verticillata* under 2 °C low-temperature stress. Different letters indicate significant differences among concentration treatments on the same sampling day (*p* < 0.05).

MDA content showed an overall increasing trend with fluctuations during stress in the control group, while BR treatment alleviated oxidative damage (Figure 3a). The 0.5 mg L^−1^ treatment was the most effective, with MDA content being significantly lower than that of the control at all sampling points (*p* < 0.05), decreasing by 29.05%, 41.96%, 23.65%, and 37.54%, respectively. The 0.1 mg L^−1^ BR treatment also significantly reduced MDA content, showing a 23.98% decrease on day 15 relative to the control, whereas 0.05 mg L^−1^ did not differ significantly from the control. SOD activity increased over time, and BR treatment further enhanced this increase (Figure 3b). The 0.05 mg L^−1^ BR treatment showed the most pronounced stimulatory effect, being significantly higher than the control at all sampling points, with a 32.44% increase on day 15. The 0.1 mg L^−1^ BR treatment increased SOD activity by 25.99% and 26.61% on days 10 and 15, while the 0.5 mg L^−1^ treatment showed no significant difference from the control. POD activity also increased under low-temperature stress (Figure 3c). The 0.05 mg L^−1^ BR treatment significantly enhanced POD activity at all sampling points (*p* < 0.05), while the 0.1 mg L^−1^ treatment showed significant enhancement by day 10. By day 15, POD activity in the control began to decline, whereas all BR treatments maintained relatively high POD activity, which was significantly higher than that in the control, with increases of 36.98%, 29.79%, and 27.70% for 0.05, 0.1, and 0.5 mg L^−1^, respectively.

### 3.4. Effect of Different BR Concentrations on Osmotic Adjustment Substances

**Figure 4:**
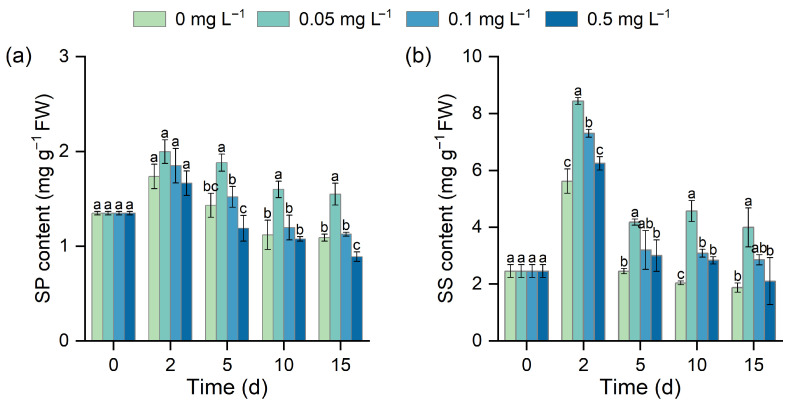
Effects of BR on SP content (**a**) and SS content (**b**) in *H. verticillata* under 2 °C low-temperature stress. Different letters indicate significant differences among concentration treatments on the same sampling day (*p* < 0.05).

SP content initially increased in all treatments and then declined over time (Figure 4a). The 0.05 mg L^−1^ BR treatment significantly increased SP content from day 5, with increases of 31.54%, 42.86%, and 42.20% on days 5, 10, and 15 (*p* < 0.05). The 0.1 mg L^−1^ treatment showed an increasing trend, but the difference was not significant, while the 0.5 mg L^−1^ BR treatment reduced SP accumulation. SS content was more sensitive to BR (Figure 4b). In the early stage of stress, all BR treatments promoted SS accumulation. On day 2, SS content in the 0.05, 0.1, and 0.5 mg L^−1^ BR treatments increased by 50.14%, 29.91%, and 11.17%, respectively, compared with the control. As the stress progressed, only the 0.05 mg L^−1^ treatment maintained a significant increase by day 15 (112.59%, *p* < 0.05), whereas the 0.1 and 0.5 mg L^−1^ treatments did not differ significantly from the control.

### 3.5. Comprehensive Evaluation of the Alleviation Effects

**Figure 5:**
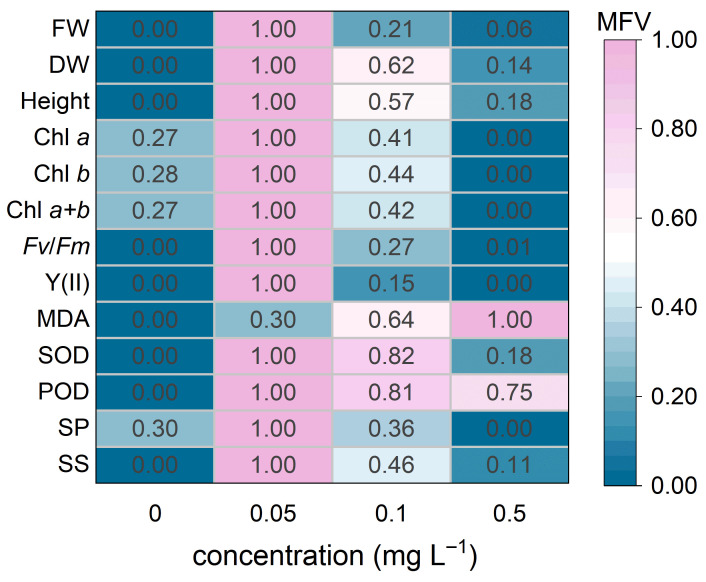
MFV evaluation of the alleviation effects of different BR concentrations on *H. verticillata* under 2 °C low-temperature stress.

Based on data collected on day 15 of low-temperature stress, MFV analysis was performed for all measured indicators (Figure 5). The 0.05 mg L^−1^ BR treatment showed the most pronounced overall alleviating effect, achieving the highest MFVs for all indicators except MDA. The 0.1 mg L^−1^ BR treatment had a relatively weaker effect, while the 0.5 mg L^−1^ BR treatment showed minimal overall alleviation, with only MDA showing a high MFV. The overall MFV values (Table 2) indicated that the effectiveness of BR treatments in improving low-temperature tolerance in *H. verticillata* decreased in the following order: 0.05 mg L^−1^ (0.95) > 0.1 mg L^−1^ (0.47) > 0.5 mg L^−1^ (0.19) > 0 mg L^−1^ (0.09).

## 4. Discussion

### 4.1. Alleviating Effects of BR on H. verticillata Under Low-Temperature Stress

Low-temperature stress is an important abiotic constraint that limits plant growth and induces morphological deterioration [33]. Fresh weight, dry weight, and plant height are widely used as direct indicators of plant growth status and stress severity. In the present study, exogenous BR application significantly improved these parameters under low-temperature stress (Figure 1), indicating that BR effectively alleviated growth inhibition and helped maintain morphological stability. These results are consistent with the findings reported by Bashir and John [34]. Previous studies have shown that BR can sustain plant growth under stress conditions by regulating growth-related gene expression [35,36]. Therefore, the growth-promoting effects observed in *H. verticillata* may be attributed to enhanced metabolic activity and the maintenance of cellular growth processes under low-temperature stress.

Photosynthesis is one of the physiological processes most sensitive to low-temperature stress [37]. Low-temperature stress generally suppresses PSII reaction center activity and electron transport efficiency, thereby reducing chlorophyll content and photosynthetic performance [38,39]. In this study, BR treatment effectively maintained chlorophyll content and stabilized *Fv*/*Fm* and Y(II) during the middle and later stages of stress (Figure 2), suggesting that BR mitigated damage to the photosynthetic apparatus under low-temperature stress. This result is consistent with the findings of Wu et al. [40]. Such protection may be related to the ability of BR to reduce ROS-mediated damage to chloroplasts and photosynthetic structures [36], as well as to improved photochemical energy utilization and reduced the risk of photoinhibition caused by excess excitation energy [41]. It should be noted that at the higher BR concentration (0.5 mg L^−1^), chlorophyll content was lower than that in the control (Figure 2a–c), indicating that the regulatory effect of BR is clearly concentration-dependent. In other words, the alleviating effect of BR appears to occur within an optimal concentration range rather than increasing continuously with increasing concentration.

Low-temperature stress also induces excessive accumulation of ROS, which can trigger membrane lipid peroxidation and impair membrane integrity. MDA is an important indicator of membrane lipid peroxidation [42], whereas antioxidant enzymes such as SOD and POD constitute a key defense system involved in ROS scavenging and oxidative damage mitigation [43,44]. Previous studies have shown that BR can enhance tolerance to low-temperature stress by regulating antioxidant defense pathways and increasing antioxidant enzyme activities [45,46]. In the present study, BR treatment significantly reduced MDA content while increasing SOD and POD activities in *H. verticillata* (Figure 3), indicating that BR may enhance antioxidant defense capacity, promote ROS scavenging, and thereby alleviate oxidative damage under low-temperature stress.

In addition to antioxidant defense, osmotic adjustment is another important mechanism by which plants cope with low-temperature stress [5]. SP and SS are important osmolytes that help maintain cellular osmotic balance, improve water retention, and protect membrane structure and protein function under stress conditions [47]. In this study, BR treatment significantly increased SP and SS contents in *H. verticillata* (Figure 4), indicating that BR may enhance osmotic adjustment capacity and thereby improve adaptation to low-temperature stress. Moreover, SP and SS may act synergistically with the antioxidant system to stabilize the intracellular environment, reduce oxidative injury, and maintain metabolic homeostasis [48].

Taken together, these results suggest that BR alleviated low-temperature stress in *H. verticillata* through the coordinated regulation of growth, photosynthetic performance, antioxidant defense, and osmotic adjustment.

### 4.2. Concentration-Dependent Differences in the Alleviating Effects of BR

The present study showed that the alleviating effects of BR on *H. verticillata* under low-temperature stress were strongly concentration-dependent. The MFV-based evaluation showed that 0.05 mg L^−1^ produced the best overall response, whereas 0.1 mg L^−1^ had a moderate effect and 0.5 mg L^−1^ provided only limited overall improvement (Figure 5; Table 2). This suggests that the beneficial effects of exogenous BR do not increase linearly with concentration. In this study, 0.05 mg L^−1^ performed best across most measured indicators, indicating that this concentration was sufficient to maintain physiological stability and enhance the overall low-temperature tolerance of *H. verticillata*. Although 0.5 mg L^−1^ was more effective in reducing MDA content, its effects on chlorophyll accumulation, fluorescence parameters, and osmotic adjustment were relatively limited, suggesting that higher BR concentrations may alleviate stress-induced membrane damage, but may not support the coordinated regulation of multiple physiological processes required for optimal stress adaptation.

This concentration dependence may be related to the dual regulatory role of BR in plants. Within an appropriate concentration range, BR can enhance stress resistance, whereas excessive application may disturb endogenous physiological balance or impose additional metabolic costs, thereby weakening its overall beneficial effect [49]. In submerged macrophytes, such sensitivity may be more pronounced because the underwater environment can affect the diffusion and uptake of exogenous hormones. Previous studies have shown that *H. verticillata* exhibits strong environmental adaptability and high metabolic sensitivity to external fluctuations [13,50], suggesting that its physiological response to exogenous hormones may vary substantially with concentration.

Overall, the optimal BR concentration should be determined by its integrated effects on multiple physiological processes rather than by the greatest improvement in a single indicator. Under the present experimental conditions, 0.05 mg L^−1^ BR achieved the best balance among photosynthetic maintenance, antioxidant defense, and osmotic regulation, and was therefore identified as the optimal concentration for improving low-temperature tolerance in *H. verticillata*.

## 5. Conclusions

BR significantly enhanced the low-temperature tolerance of *H. verticillata* in a concentration-dependent manner. Among the tested treatments, 0.05 mg L^−1^ BR showed the strongest overall effect, as reflected by improved growth, maintained photosynthetic performance, enhanced antioxidant defense, and increased osmotic adjustment under 2 °C low-temperature stress. In contrast, higher BR concentrations were less effective overall. These results indicate that 0.05 mg L^−1^ is the most effective BR concentration among the tested treatments under the present experimental conditions and suggest that BR-mediated low-temperature tolerance in *H. verticillata* is associated with the coordinated regulation of multiple physiological processes. These findings provide a physiological basis for the application of BR in the winter conservation of submerged macrophytes and for the development of strategies to improve low-temperature tolerance during aquatic ecological restoration.

## Figures and Tables

**Table 1:** Effects of different BR concentrations on the biomass growth rate of *H. verticillata* under 2 °C low-temperature stress.

BR Concentration (mg L^−1^)	FW BGR (%)	DW BGR (%)
0.00	−7.58	−13.64
0.05	7.41	5.93
0.10	−4.48	−1.57
0.50	−6.62	−10.95

**Table 2:** Overall MFV and ranking of the alleviation effects of different BR concentrations on *H. verticillata* under 2 °C low-temperature stress.

BR Concentration (mg L^−1^)	Overall MFV	Ranking
0.00	0.09	4
0.05	0.95	1
0.10	0.47	2
0.50	0.19	3

## Data Availability

Data are available from the corresponding author upon reasonable request.
